# Platinum Atoms Dynamics on the Surface of Hexagonal Boron Nitride Containing Vacancy Defects

**DOI:** 10.1021/acsami.5c22977

**Published:** 2026-01-27

**Authors:** Sadegh Ghaderzadeh, Ilya Popov, Wolfgang Theis, Jesum Alves Fernandes, Andrei N. Khlobystov, Elena Besley

**Affiliations:** † School of Chemistry, 6123 University of Nottingham, University Park, Nottingham NG7 2RD, UK; ‡ School of Physics and Astronomy, 1724 University of Birmingham, Edgbaston B15 2TT, UK

**Keywords:** single-atom catalysts, hexagonal boron nitride, defect engineering, ab initio methods, kinetic nucleation theory, magnetron sputtering

## Abstract

Single-atom catalysts (SACs) have transformed the field of heterogeneous catalysis by enabling efficient utilization of metal atoms and enhancing the selectivity and activity of chemical reactions. The propensity of metal atoms to aggregate into nanoclusters complicates the consistent production of SACs and creates challenges in understanding their interactions with naturally defected supports. Based on the example of platinum SACs on hexagonal boron nitride, this study combines ab initio computational methods with kinetic nucleation model to propose a route to controlled fabrication of SACs through defect engineering. It shows that diffusion barriers obtained for an isolated SAC on pristine surface do not represent realistic growth conditions and highlights the importance of accounting for collective atomic behavior when modeling nucleation and growth processes. The study extends the classical Volmer–Weber mechanism of nanocluster growth to account also for the presence of surface vacancy defects and predicts the values of the single atom-to-nanocluster ratio as a function of the surface defect density and platinum loading. The effect of ambient oxygen on platinum SACs formation has been examined to investigate its role in hindering metal interactions with defects and promoting clustering.

## Graphical abstract

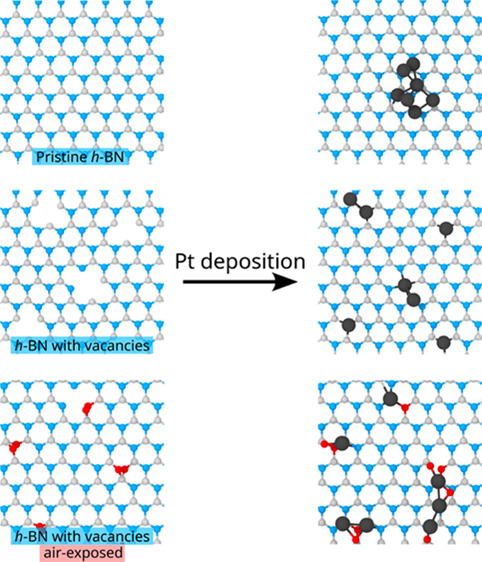

## Introduction

The ever-increasing demand of chemical industry for catalysts based on precious metals and sparse natural resources has stimulated extensive research efforts aimed at developing innovative approaches to catalysis with low utilization of metals.^1–3^ Single-atom catalysts (SACs)—individual metal atoms anchored on solid supports—have emerged as a promising frontier in heterogeneous catalysis and showed an unprecedented performance in chemical reactions with significant environmental relevance.^3^ For specific types of chemical reactions, SACs can offer enhanced catalytic activity and selectivity, compared to traditional nanoparticle catalysts,^4–7^ due to the unique metal coordination structure and interactions with the support. Several approaches to fabrication of stable SACs have been proposed;^8–21^ however, these methods have their limitations, particularly in achieving high loading of single-metal atoms while avoiding the excessive formation of metal nanoparticles or clusters. A reliable control of the dispersion and stability of SACs during chemical reactions is also crucial to their performance. For example, stability against sintering and leaching is of the utmost importance in maintaining the activity and selectivity of SACs.

Addressing these challenges requires comprehensive understanding of the interactions between metal atoms and surfaces, and the importance of surface defects in designing single atom and metal cluster catalysts has been acknowledged previously.^13,19,22^ Our earlier work^23^ proposed to use kinetic nucleation theory to explain and refine physical vapor deposition experiments.^24,25^ It showed that the nature and size of metal species formed on a substrate can be tuned through only a small number of experimental parameters,^23^ thus enabling deposition of a variety of transition metals (and their combinations) and a broader flexibility in the synthesis of SACs.^24,26–28^ In this study, we combine density functional theory (DFT) and ab initio molecular dynamics (AIMD) simulations with the kinetic nucleation model^23^ to propose a route to a controlled fabrication of SACs through defect engineering. We show that the formation of metal nanoparticles or clusters can be mitigated through stabilization of metal atoms on surface defect sites, which is an essential step in achieving a single-atom dispersion. We further demonstrate that diffusion barriers required in kinetic nucleation models and derived for an isolated atom on defect-free surface neglect the collective behavior of SACs in deposition experiments and can lead to inaccurate predictions of SACs mobility. This highlights the need to incorporate many-body effects into models of nucleation and growth.

We focus on investigating the interactions of Pt atoms with pristine and defective hexagonal boron nitride, *h*-BN, and calculate the binding energy of a single Pt atom and Pt clusters on pristine and defective *h*-BN as well as the transition states and energy barriers separating Pt atoms on a pristine *h*-BN site from various defective and defect-occupied sites. Our DFT and AIMD calculations show that defect concentration plays a key role in Pt dispersion on the *h*-BN surface, which determines the metal surface area available for catalysis. We show that Pt nucleation on pristine *h*-BN follows the island-growth mechanism, known as the Volmer–Weber growth (see ref 29 and references therein), in which adatom–adatom interactions are stronger than the interactions of an adatom with the surface, leading to the formation of three-dimensional clusters or islands. In the case of pristine *h*-BN, Pt atoms favor clusterization over dispersion. However, dispersion of Pt atoms on defect sites, vacancies in particular, has a significant stabilization effect and makes SACs energetically more favorable. Previous DFT and AIMD studies on stabilization of isolated metal atoms on supports with vacancy defects confirm some of these conclusions, in particular the studies of Pt atoms on *h*-BN (B-vacancy),^30^ Pd atoms on *h*-BN (B- and N- vacancies),^31^ Fe atoms on MoS_2_ (S-vacancy),^32^ Rh atoms on CoO (O-vacancy),^33^ Co atoms on graphene (C-vacancy),^34^ Au atoms on graphene (C-vacancy),^25,35^ and CeO_2_ (O-vacancy),^36,37^ as well as a range of metal atoms on FeO_*x*_ (O-vacancy).^38^

Our study suggests a delicate interplay between concentration of vacancies and mobility of Pt atoms. For the case of Pt on *h*-BN, we predict the single-atom-to-nanocluster (SA:NC) ratio as a function of the surface defect density and Pt loading. Our AIMD simulations also describe the impact of air exposure on the defect-mediated fabrication of SACs. These results can be used in guiding physical vapor deposition experiments aimed at enhancing the catalytic activity of metal-decorated surfaces through the control of the SA:NC ratio.

## Computational Methods

Spin-polarized DFT calculations of the binding energies and spin-polarized DFT-based climbing-image nudged elastic band (CI-NEB) calculations of transition states^39^ have been performed using the plane-wave projector augmented—wave (PAW) method available in the Vienna Ab initio Simulation Package (VASP).^40,41^ The structures were relaxed using the Perdew–Burke–Ernzerhof (PBE) exchange–correlation functional^42^ with a force tolerance of 0.005 eV A^–1^ and the electronic convergence criteria of 10^–6^ eV. The energy cutoff was set to 450 eV, and a gamma-point-centered Monkhorst–Pack k-point grid of 3 × 3 × 1 was used to sample the Brillouin zone. van der Waals interactions were taken into account using the DFT-D3 method of Grimme.^43,44^ Pristine *h*-BN supercell contained 144 boron and 144 nitrogen atoms for calculations of the binding energies and 36 boron and 36 nitrogen atoms for calculations of transition states. The total number of electrons treated explicitly in VASP calculations is 3 electrons (B), 5 electrons (N), and 10 electrons (Pt).

In the presence of vacancy, the binding energy was calculated as follows:

$$E_{b}=(E_{V-\mathrm{Pt}}-E_{V}-nE_{\mathrm{Pt}})/n$$ 1

where *E*_V–Pt_ is the total energy of the system (Pt atom on top of *h*-BN layer containing vacancy defect), *E*_V_ is the total energy of *h*-BN layer containing vacancy, *E*_Pt_ is the total energy of an isolated Pt atom in the simulation cell, and *n* is the number of Pt atoms.

Ab initio molecular dynamics calculations have been performed using the CP2K^45^ package with PBE functional^42^ and a hybrid Gaussian/plane-wave scheme (GPW).^46^ Valence electrons were expanded in double-Gaussian basis sets with one polarization function (DZVP) optimized for multigrid integration,^47^ while the core electrons and nuclei were described by Goedecker–Teter–Hutter (GTH) pseudopotentials.^48^ In AIMD simulations, dispersion interactions were accounted for using DFT-D3 level of theory.^43^ Four multigrids and a cutoff of 300 Ry have been used. Benchmarking tests against a tighter convergence with the cutoff of 400 Ry have been shown to change the binding energy by 0.001 eV. Hence, the cutoff of 300 Ry was considered to be a reasonable compromise between the accuracy and cost of the obtained results. The simulation cell contained 80 boron and 80 nitrogen atoms in the pristine *h*-BN system. The total number of electrons treated explicitly in CP2K calculations is 3 electrons (B), 5 electrons (N), and 18 electrons (Pt). The geometries of all of the structures were optimized.

An MD time step of 0.2 fs was used in AIMD simulations, and the temperature control was achieved using the canonical sampling through velocity rescaling (CSVR) thermostat, as implemented in CP2K. The AIMD simulations were conducted within the NVT ensemble at a temperature of 300 K. Three distinct *h*-BN environments were studied: pristine, Ar-treated, and Ar-treated, followed by exposure to air. Prior to metal deposition, *h*-BN structures were thermally equilibrated at 300 K by using the NVT ensemble. In all three cases, Pt atoms were deposited sequentially, one at a time, with the kinetic energy of 8 eV at identical locations. Following the deposition of each Pt atom, the system was maintained at 300 K for approximately 1–5 ps until no further structural changes were observed, i.e., the metal atom settled into a local minimum on the defective substrate, thereby ensuring an equilibrium state. For consistency, comparable equilibration times were applied to the pristine *h*-BN sheet, which resulted in the formation of metal cluster formation. The atomic visualizations are created using the Open Visualization Tool (OVITO).^49^

## Results and Discussion

The dynamics and distribution of platinum on the *h*-BN surface in physical vapor deposition experiments has been studied using kinetic nucleation model (KNM),^23^ which incorporates diffusion and interfacial attachment of Pt atoms as well as cluster growth at the time and length scales not accessible to ab initio molecular dynamics. The growth mechanism, as described by KNM, is based on quantitative estimations of the strength of interactions between individual metal atoms and the substrate with the use of DFT. We compare the DFT binding energies involving several Pt atoms in clustered and dispersed forms on pristine *h*-BN to show the thermodynamic preference for clustering over dispersion (Figure S1 of the Supporting Information). This indicates that Pt nucleation on pristine *h*-BN follows the island-growth (Volmer–Weber) mechanism, where Pt–Pt interactions are stronger than Pt–substrate interactions.

The classical Volmer–Weber mechanism does not account for surface defects such as vacancies. However, Pt atom behavior on *h*-BN is substantially different in the presence of vacancies on the substrate. For example, several Pt atoms dispersed on *h*-BN layer containing vacancy defects (both B and N vacancies) have lower value of the total energy than clusters formed on the same defective support (Figures S2 and S3 of the Supporting Information), indicating that, unlike in the case of pristine *h*-BN, clusterization is energetically less favorable than dispersion on vacancy defects in *h*-BN. In this case, Pt atoms preferentially occupy available defect sites in order to reduce the total energy. The interaction of individual Pt atoms with the substrate is strongly influenced by the presence of vacancies. In pristine *h*-BN, the most stable adsorption site is nitrogen atom with the binding energy of Pt atom to nitrogen site of −1.84 eV. The binding is much stronger on vacancy defects in *h*-BN; the binding energy of Pt atom to divacancy is −7.95, −7.30 eV to N vacancy, and −7.48 eV to B vacancy. Experimentally observed stable, isolated Pt atoms on *h*-BN, as seen in AC-STEM images,^23^ can be attributed to point defects which act as strong binding sites and enable effectively irreversible attachment. As native point defects are inevitably present in any two-dimensional material, a comprehensive kinetic model must incorporate their effects.

At a given temperature, the relative rates of competing nucleation processes in an island-growth mechanism, modified to include point defects on the surface, are governed by the corresponding energy barriers. These energy barriers also determine the KNM predictions of the SA:NC ratio for a given Pt loading, vacancy concentration, and temperature. The climbing-image nudged elastic band (CI-NEB) method has been used to calculate the energy barriers for the following elementary processes: Pt atom migrating between two adsorption N sites on pristine *h*-BN (Figure S4 of the Supporting Information); Pt atom migrating from a N site to a B vacancy (Figure 1a); Pt atom migrating from a N site to a N vacancy (Figure 1b); Pt atom migrating from an N site to join another Pt atom bonded to a B vacancy (Figure 1c); and Pt atom migrating from an N site to join another Pt atom bonded to an N vacancy (Figure 1d).

**Figure 1:**
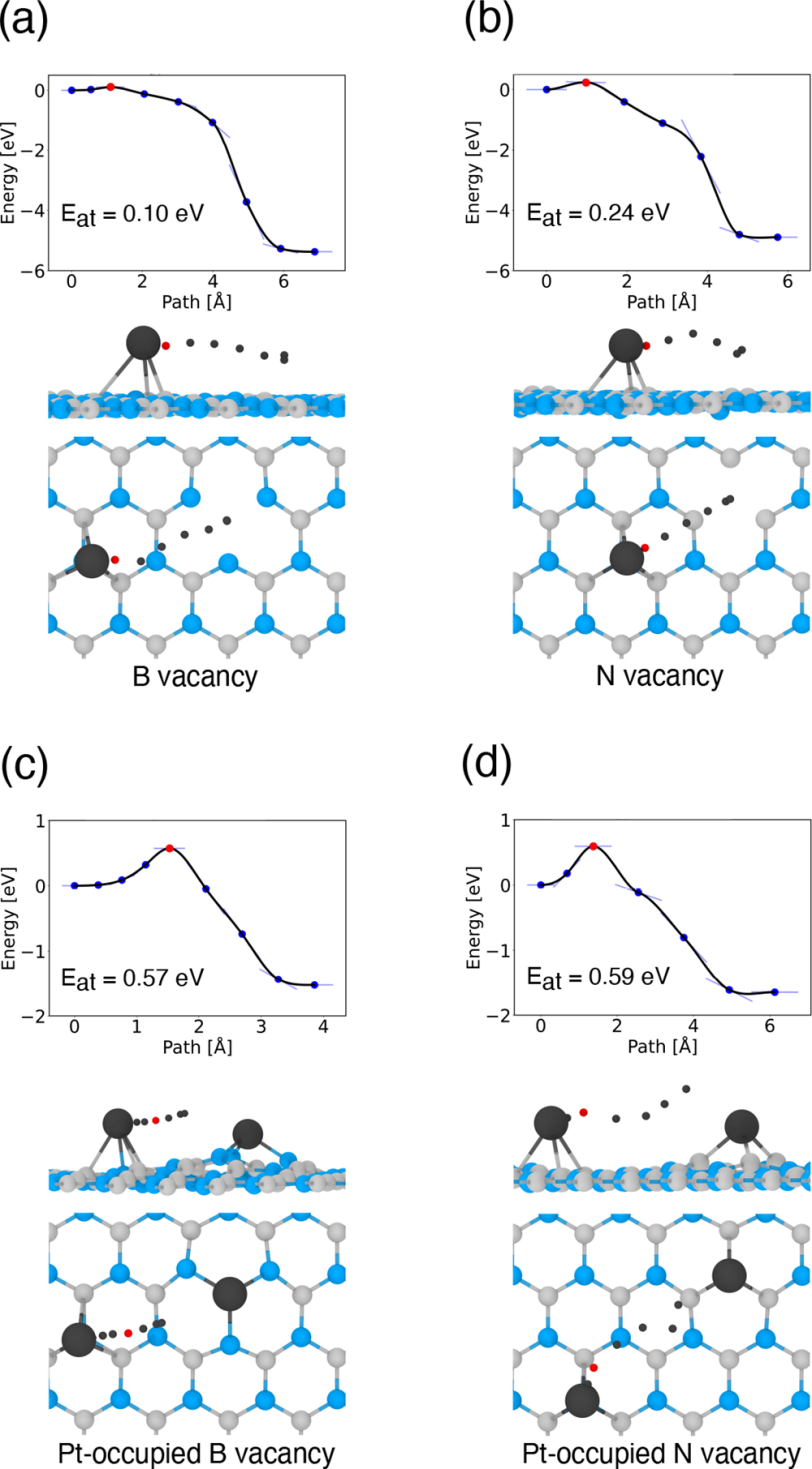
DFT-D3 CI-NEB energy barriers to Pt atom migration from an N site on *h*-BN to a B vacancy (a), an N vacancy (b), a Pt-occupied B vacancy (c), and a Pt-occupied N vacancy (d). The transition state is indicated by red circle; black circles show the path undertaken by the Pt atom. Blue: nitrogen, gray: boron, and black: platinum.

The initial position of the migrating Pt atom was chosen to be the nearest energetically favorable site (i.e., above N atom) from which the Pt atom does not spontaneously recombine during structural relaxation with the target site. The vacancy concentration in Figure 1 corresponds to the value of 5 × 10^13^ vacancies/cm^2^ which is typical for ion beam irradiation experiments. Recent experimental work^50^ reports a comparable value for the vacancy concentration in graphene, 4 × 10^13^ vacancies/cm^2^, achieved at the lowest irradiation dose of 1 × 10^14^ ions/cm^2^. Previous theoretical study^51^ also showed that similarly high, predominantly single-vacancy concentrations can be generated in *h*-BN by ion irradiation with the kinetic energy just above the displacement threshold (e.g., ≈50 eV) and fluxes of the order of 5 × 10^14^ ions/cm^2^.

The CI-NEB calculations (Figure 1) predict a low energy barrier of 0.10 eV to recombination of Pt atom with boron vacancy, while recombination of Pt atom with nitrogen vacancy requires overcoming a greater barrier of 0.24 eV. An increase in the energy barrier is due to the fact that in reaching nitrogen vacancy the path of Pt atom goes over the energetically less favorable boron site (Figure 1b). In contrast, reaching a boron vacancy involves migration over the favorable N site, and it results in lowering the energy barrier (Figure 1a). For comparison, the migrating Pt atom encounters significantly higher energy barriers (0.57–0.59 eV) when attempting to join a Pt-occupied vacancy site (either on B or N vacancy). While the first Pt atom migrating to a vacancy site encounters a relatively low energy barrier, a subsequent adatom must overcome a higher barrier to reach the same site as the second Pt atom gets detached from the surface and remains elevated above the target Pt atom, which already occupies the vacancy (Figure 1c,d).

The DFT-D3 diffusion barrier between two N sites was calculated as *E*_diff_ = 0.747 eV (Figure S4a of the Supporting Information) which is in excellent agreement with the previously reported value of 0.75 eV.^52^ This energy barrier is higher than the barriers of attachment to vacancy or Pt-occupied vacancy sites. The diffusion coefficient, *D*, of Pt adatoms on pristine *h*-BN surface can be defined as^53^

$$D=\frac{a^{2}\nu_{0}}{z}\exp\left(-\frac{E_{\mathrm{diff}}}{RT}\right)$$ 2

where *a* is the distance between the nearest adsorption minima in *h*-BN lattice, ν_0_ ≈ 10^13^ s^–1^ is the vibrational frequency (see for example ref 54), and *E*_diff_ is the diffusion barrier. The parameter *z* denotes the number of symmetry equivalent hopping sites available to the adatom on the *h*-BN lattice. As the adsorption minimum site for Pt atom on *h*-BN is on top of N, *a* = 0.2515 nm and *z* = 6, giving the value of the pre-exponential factor of 10^–7^ m^2^ s^–1^, and *D*_DFT‑D3_ = 2.4 × 10^–20^ m^2^ s^–1^ at room temperature. Mean squared displacement for two-dimensional surface diffusion on a lattice with six-coordinated adsorption sites can be estimated as

$$\left\langle r^{2}\right\rangle =6Dt$$ 3

For *t* = 1 s, eq 3 yields ⟨*r* ^2^⟩ = 0.14 nm^2^ which corresponds to root-mean-square displacement of $\sqrt{\left\langle r^{2}\right\rangle }\approx 0.37\mathrm{nm}$ . Thus, DFT predicts that a single Pt atom on pristine *h*-BN is effectively immobile at room temperature. However, the presence of other Pt atoms and point defects on the *h*-BN surface significantly alters the diffusion landscape and its energetics, facilitating the migration of Pt atoms by lowering the diffusion barrier locally.

To confirm the effect of surface adatoms on the reduction of the diffusion coefficient of the migrating metal atom, we carried out two types of AIMD calculations: (1) migration of a single Pt atom on pristine *h*-BN surface at room temperature and (2) deposition of several Pt atoms at random positions on the surface followed by the equilibration of the system at room temperature. As expected from the high value of the calculated DFT-D3 diffusion barrier, a single Pt atom on pristine *h*-BN does not possess high mobility at room temperature (≈0.37 nm s^–1^) and it does not diffuse from its original position (see Movie S1 in the Supporting Information). However, the presence of other Pt atoms lowers the effective diffusion barrier leading to higher mobility of metal adatoms on *h*-BN surface even at room temperature and it results in clusterization (Figure 2 and Movie S2 in the Supporting Information). Note that the most stable form of an isolated Pt_7_ cluster in gas phase is 3D-coupled tetragonal pyramid.^55^ However, when supported on surfaces, the accessible isomers can vary with temperature and, especially, in the presence of surface defects.

**Figure 2:**
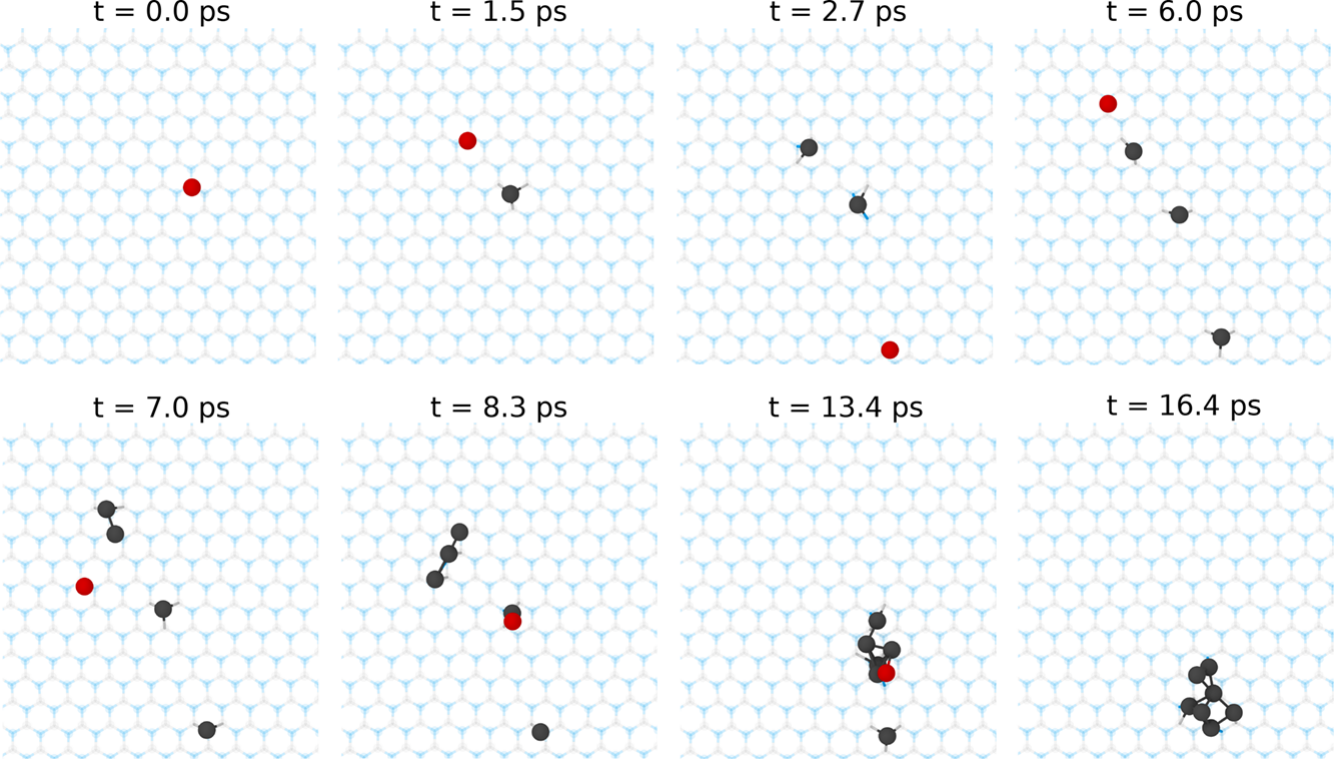
Ab initio molecular dynamics simulation of the coalescence of several Pt atoms landed sequentially at random locations on pristine *h*-BN at *T* = 300 K. The initial kinetic energy of landing is taken to be 8 eV to ensure soft landing. The landing position for each Pt atom is shown in red. For further details, see Movie S2 in the Supporting Information. Blue: nitrogen, gray: boron, black, and red: platinum.

The smaller effective diffusion barrier, compared to the DFT values calculated on the pristine surface, is supported by the analysis of physical vapor deposition experimental data from which the experimental value for the diffusion coefficient, *D*_exp_, can be extracted.^23,56^ The dependence of the experimentally observed surface density of nanoclusters on the diffusion coefficient calculated for the experimental value of the vacancy concentration in *h*-BN^23^ (*N*_d_ = 0.1 nm^–2^) is shown in Figure S4 of the Supporting Information. This graph allows us to estimate *D*_exp_ in the range of 1.0 × 10^–13^–2.2 × 10^–15^ m^2^ s^–1^, and the diffusion barrier, *E*_diff_^exp^, ranging from 0.36 to 0.45 eV (see details in Figure S4 in the Supporting Information). The values of the diffusion coefficient extracted from experiment are 5–6 orders of magnitude larger than DFT estimations for an isolated Pt atom on pristine surface. This apparent discrepancy is consistent with the greatly enhanced mobility of the Pt atom shown in ab initio molecular dynamics simulations of multiple atom landing events (Figure 2 and Movie S2 in the Supporting Information). While DFT values for the diffusion barriers do not fully capture the complexity of experimental conditions, DFT estimates are generally reliable for the attachment barriers where the local environment in simulations and experiments are comparable. Additionally, the AIMD calculations indicate that surface diffusion of Pt often involves forming dimers, trimers, and small clusters which are not accounted for in KNM.

The Volmer–Weber KNM, augmented with additional equations accounting for the nucleation and formation of single atoms at the point defects,^23^ help to investigate the Pt nucleation process in the presence of vacancies in *h*-BN. This model contains a set of differential equations describing time evolution of the surface density of Pt nanoclusters (see Supporting Information S2for more details). In the KNM, when metal atoms come into contact with a surface, they can diffuse and coalesce to form a nucleus through a process known as homogeneous nucleation. Alternatively, they bind to pre-existing defects on the surface, resulting in the formation of stationary stable single atoms. These immobilized atoms then serve as nucleation sites for further attachments leading to heterogeneous nucleation on the surface. The resulting ratio of stable single atoms to nanoclusters, SA:NC, is the combined effect of these two processes.

To describe the relative rates of the homo- and heterogeneous nucleation processes, we first consider an isolated attachment center (adatom, nanocluster, vacancy, or metal atom occupying a vacancy) on the surface. As demonstrated by the CI-NEB calculations (Figure 1), the attachment center forms a capture region through a local perturbation of the potential energy surface, which can be characterized by the energy barrier to attachment. We describe this region as a circle with the radius *r*_d_ outside which adatoms diffuse by hopping from one adsorption site to another as defined by the diffusion coefficient, *D*, in eq 2. In the diffusion region, a stationary solution for the surface concentration of adatoms *f*(*r*) satisfies the Fick’s second law in polar coordinates

$$\frac{D}{r}\frac{d}{dr}\left(r\frac{df\left(r\right)}{dr}\right)=0$$ 4

with the boundary conditions *f*(*r*_d_) = *n̅*_1_ and *f*(*l*_d_) = *n*_1_. Here, *n*_1_ is the average concentration of adatoms on the surface, *n̅*_1_ is the concentration of adatoms on the border of the capture region, and *l*_d_ is the screening distance defined as a distance from the attachment center at which the surface concentration profile *f*(*r*) reaches its average value *n*_1_. Solution to eq 3 yields the following rate of adatom diffusion to the capture region^57^

$$j_{\mathrm{diff}}=\frac{2\pi }{\ln(l_{d}/r_{d})}D[n_{1}-{\overset{¯}{n}}_{1}]=\sigma_{d}D[n_{1}-{\overset{¯}{n}}_{1}]$$ 5

At the same time, the flux of atoms attaching from the border of the capture region to the attachment center is given by

$$j_{\mathrm{at}}=r_{d}\sqrt{\frac{\pi RT}{2M}}\exp\left(-\frac{E_{\mathrm{at}}}{RT}\right){\overset{¯}{n}}_{1}$$ 6

where *M* is the molar mass of the adatoms (Pt in this case). The condition of continuity of the flux at the border of the capture region (*j*_diff_ = *j*_at_) allows us to exclude the unknown concentration *n̅*_1_ and obtain the following equation for the rate of attachment to a single attachment center

$$j_{d}=\sigma_{d}D{\left[1+\chi_{d}\exp\left(\frac{E_{\mathrm{at}}-E_{\mathrm{diff}}}{RT}\right)\right]}^{-1}n_{1}$$ 7

where the dimensionless parameter

$$\chi_{d}=\frac{a^{2}\nu_{0}}{zr_{d}\ln(l_{d}/r_{d})}\sqrt{\frac{8\pi M}{RT}}$$ 8

has the value of 1.5–2 for a Pt atom on *h*-BN at room temperature. Taking into account the surface concentration of attachment centers *n*_d_, one obtains the final expression for the rate of attachment

$$v_{d}=j_{d}n_{d}=\beta_{d}\sigma_{d}Dn_{1}n_{d}$$ 9

where the scaling parameter β_d_ is defined as

$$\beta_{d}={\left[1+\chi_{d}\exp\left(\frac{E_{\mathrm{at}}-E_{\mathrm{diff}}}{RT}\right)\right]}^{-1}$$ 10

Equation 10 distinguishes two regimes of nucleation at a given attachment center: (1) a diffusion-limited regime, where *E*_at_ ≪ *E*_diff_ and β_d_ ≈ 1; and (2) an attachment limited regime, where an additional barrier suppresses nucleation relative to the diffusive case, i.e. *E*_at_ ≥ *E*_diff_ and β_d_ < 1. The markedly low attachment barriers of Pt atoms to unoccupied B and N vacancies (Figure 1a,b), compared to both the DFT and experimental values of the diffusion barriers, indicate that attachment to unoccupied vacancies is limited by diffusion and consistent with the diffusion-limited regime (1). By contrast, for vacancies already occupied by Pt atoms (Figure 1c,d), the higher attachment barriers relative to the experimental diffusion coefficient imply nucleation deactivation (regime 2), resulting in dispersed single atoms coexisting with nanoclusters on the *h*-BN surface. This corresponds to β_d_ ≪ 1, which suppresses further nucleation on Pt-occupied vacancies and favors stabilization of single atoms. We note that using the DFT value for the diffusion barrier, which exceeds the attachment barriers, would instead yield a diffusion limited scenario.

The KNM results shown in Figure 3a suggest that the SA:NC ratio depends not only on the chemical nature of defects, as reflected in the value of β_d_, but also on the initial concentration of defects, *N*_d_. Note that *N*_d_ is the *initial* concentration of vacancy defects, before metal deposition, whereas *n*_d_ is the concentration of vacancy defects as a function of time, which decreases with time as the deposition continues and vacancies get occupied. For the same value of β_d_, a higher concentration of defects allows a greater degree of atomization. Accordingly, the most effective strategy for achieving a high ratio of single atoms to nanoclusters is to introduce defects with a small value of β_d_, such as B and N vacancies, at the highest possible concentration. In Figure 3a, the region of the highest SA:NC ratio is shown in green. Single B or N vacancies on the *h*-BN surface can be created effectively using Ar plasma treatment prior to metal deposition. However, these vacancies are highly reactive and exposure to air can saturate them rendering the defects ineffective for anchoring metal atoms. Therefore, defect engineering and metal deposition must be carried out in vacuum prior to exposing samples to air.

**Figure 3:**
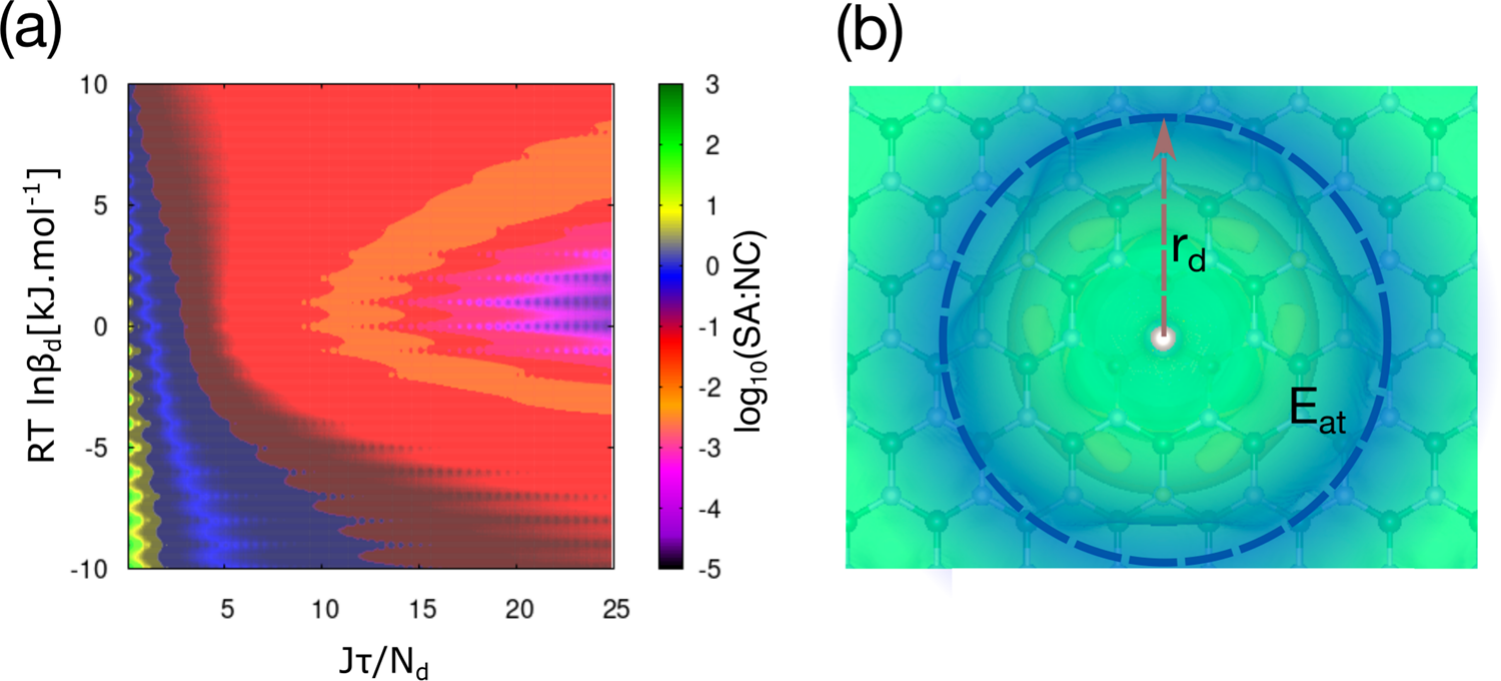
(a) Decimal logarithm of the SA:NC ratio as a function of the scaling parameter, β_d_, and the ratio of the loading of Pt atoms, *J*τ, to the initial surface concentration of defects, *N*_d_. The narrow, green-yellow region indicates physical vapor deposition conditions, which lead to the predominant existence of single Pt atoms on *h*-BN surface; the blue area corresponds to the coexistence of nanoclusters and single atoms; the large red-purple area indicates the predominant existence of nanoclusters. (b) Difference in the local potential caused by the presence of Pt adatom on N site of *h*-BN, which outlines the region of attractive Pt–Pt interactions defined by the energy barrier to attachment, *E*_at_, and radius, *r*_d_.

The impact of air exposure on metal cluster formation at vacancy defects in *h*-BN is poorly understood. Yet, understanding how atmospheric conditions influence the dynamics of metal–support interactions is crucial as heterogeneous catalysts are typically handled in air during experiments. We carried out AIMD calculations to compare the capture of Pt atoms on *h*-BN with vacancies before and after exposure to air. Oxygen molecules were placed at the vacancy sites on the *h*-BN surface, and the system was thermally equilibrated at room temperature. In both cases, Pt atoms were deposited at the identical locations on the surface. In the presence of oxygen, some Pt atoms near defect sites did not bind but instead diffused away and coalesced with other Pt atoms (see, e.g., the red trace in Figure 4 and Movie S3 in the Supporting Information). By contrast, in the absence of oxygen, the Pt atom at the same location immediately bonded to the nearest available vacancy (Figure 5 and Movie S4 in the Supporting Information). This behavior indicates that oxygen molecule passivates surface vacancies and prevents them from serving as anchoring sites for Pt atoms. Our DFT calculations further corroborate this conclusion: in the presence of oxygen, the binding energy of Pt atom decreases from −7.95 to −4.14 eV at divacancy, from −7.48 to −4.25 eV at B vacancy, and from −7.30 to −3.63 eV at N vacancy. Thus, avoiding air exposure is essential for fabricating stable single-metal atoms on the surface.

**Figure 4:**
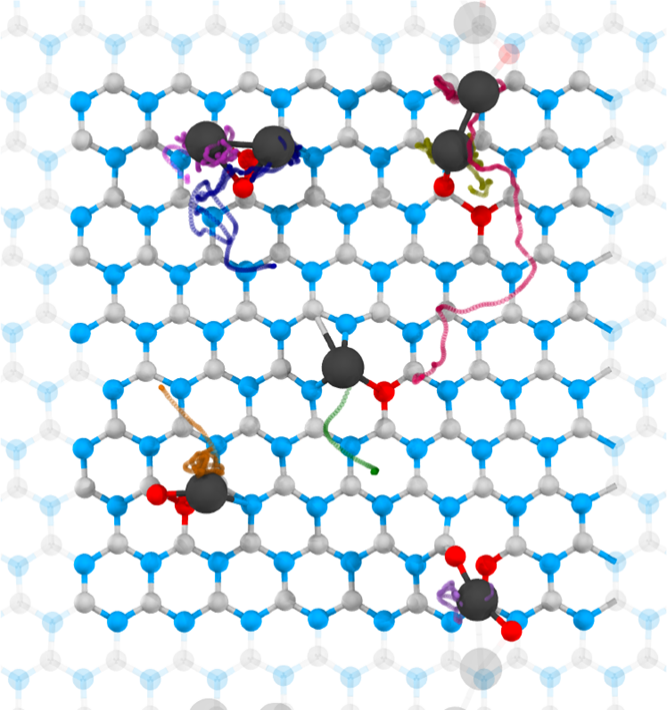
Ab initio molecular dynamics simulation (after 12.4 ps) of the deposition of Pt atoms on *h*-BN at *T* = 300 K in the presence of oxygen (red) on the vacancy sites. The initial kinetic energy of landing is taken to be 8 eV to ensure soft landing. Pt atoms (black) are deposited at the same locations as on pristine surface. For further details, see Movie S3 in the Supporting Information. Blue: nitrogen, gray: boron, black: platinum, and red: oxygen.

**Figure 5:**
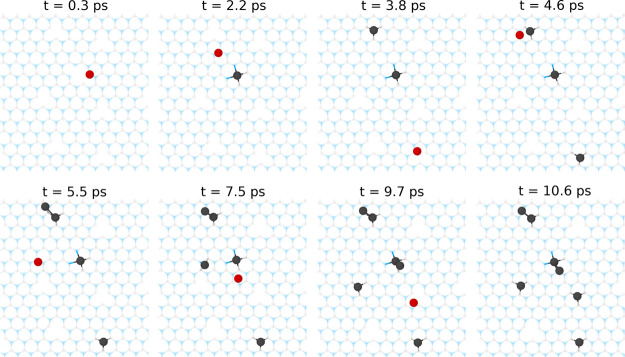
Ab initio molecular dynamics simulation (after 10.6 ps) showing the stabilization of several Pt atoms landed sequentially at random initial locations on defective *h*-BN containing single vacancies at *T* = 300 K. The initial kinetic energy of landing is taken to be 8 eV to ensure soft landing. The landing position for each Pt atom is shown in red. For further details, see Movie S4 in the Supporting Information. Blue: nitrogen, gray: boron, and black: platinum.

## Conclusions

The integrated ab initio and kinetic nucleation framework demonstrates that careful control of surface defect density and deposition parameters enables tuning of the SA:NC ratio. By explaining the interplay between point defect (vacancy) concentration, deposition time, and metal flux, we provide a practical guide for achieving stable single-atom dispersions in physical vapor deposition experiments. The Pt on *h*-BN case study reveals that the DFT diffusion barriers derived for an isolated Pt atom on pristine surface do not represent the complexity of experimental deposition environments. Ab initio molecular dynamics simulations highlight how nearby adatoms, defect sites, and air-induced surface modifications affect Pt atom mobility and nanocluster formation. Our findings underscore the importance of in situ defect generation prior to metal atom deposition for maximizing the stability of single-atom catalysts. These conclusions are transferable to other metals and supports, provided the strength of metal atom binding to vacancy on surface exceeds the strength of the interaction between metal atoms on the same surface. This condition is typically satisfied at reactive vacancy sites, and it enables the fabrication of stable single-atom dispersions for applications in catalysis and quantum technologies. It makes it also possible to predict the optimal metal deposition conditions and defect-engineering strategies to suppress unwanted island growth. Further integrating our predictions with real-time surface diagnostics and catalytic testing will refine fabrication protocols under realistic operational conditions. Extending this approach to dynamic reaction environments and multicomponent alloys promises to accelerate the development of high performance, low-loading catalysts for energy, environmental, and quantum applications.

## Supplementary Material











## Notes

Relaxed DFT structures of seven Pt atoms on pristine and defective *h*-BN (containing B- and N-vacancies); NEB diffusion barrier for Pt adatoms on pristine *h*-BN; kinetic model results showing dependence of the surface density of Pt nanoclusters on the diffusion coefficient of Pt adatoms on *h*-BN; and full set of differential equations for the Volmer–Weber nucleation model including defect-assisted growth (PDF)
Movie S1: Ab initio molecular dynamics simulation of the dynamics of a single Pt atom on pristine *h*-BN surface at T = 300 K (MP4)
Movie S2: Ab initio molecular dynamics simulation of the coalescence of several Pt atoms landed sequentially at random locations on pristine *h*-BN at T = 300 K (MP4)
Movie S3: Ab initio molecular dynamics simulation of deposition of Pt atoms on *h*-BN at T = 300 K in the presence of oxygen (red) on the vacancy sites in *h*-BN (MP4)
Movie S4: Ab initio molecular dynamics simulation showing the stabilization of several Pt atoms landed sequentially at random initial locations on defective *h*-BN containing single vacancies at T = 300 K (MP4)
