# Supplementary material for: Platinum Atoms Dynamics on the Surface of Hexagonal Boron Nitride Containing Vacancy Defects
Source: ACS Appl Mater Interfaces. 2026 Jan 27;18(5):9216–24. doi: 10.1021/acsami.5c22977 (PMC12903112; doi:10.1021/acsami.5c22977)
Supplement: Supplementary file 1 [file am5c22977_si_001.pdf]

Supporting Information: Platinum atoms dynamics on the surface of hexagonal boron nitride containing vacancy defects

Sadegh Ghaderzadeh,† Ilya Popov,† Wolfgang Theis,‡ Jesum Alves Fernandes,† Andrei N. Khlobystov,† and Elena Besley*,†

†*School of Chemistry, University of Nottingham, University Park, Nottingham NG7 2RD, UK*

‡*School of Physics and Astronomy, University of Birmingham, Edgbaston, B15 2TT, UK*

E-mail: Elena.Besley@nottingham.ac.uk

S1: Supporting information for DFT calculations

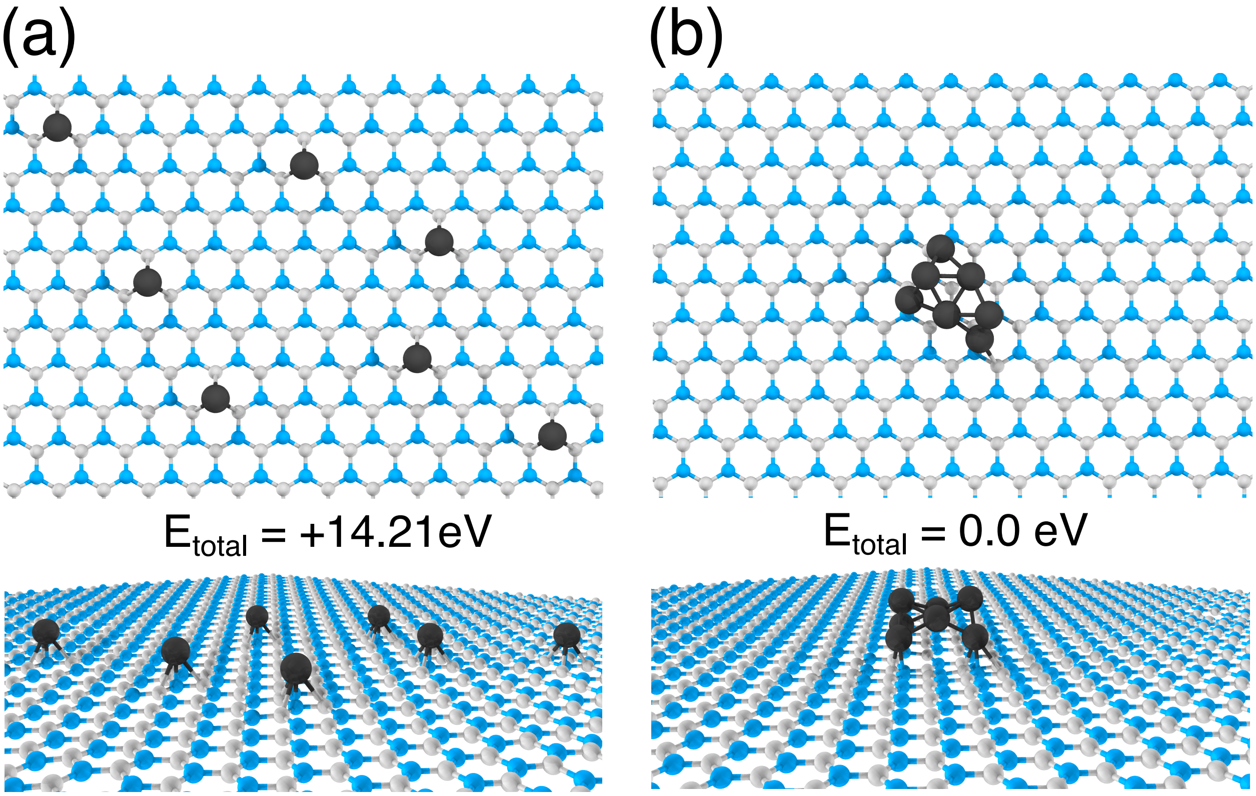

Figure S1: Relaxed structures of seven Pt atoms on pristine *h*-BN. The clustered configuration is 14.21 eV lower in energy than the dispersed form, making it more energetically favourable. Top-view (top panels) and side-view (bottom panels) images are shown.

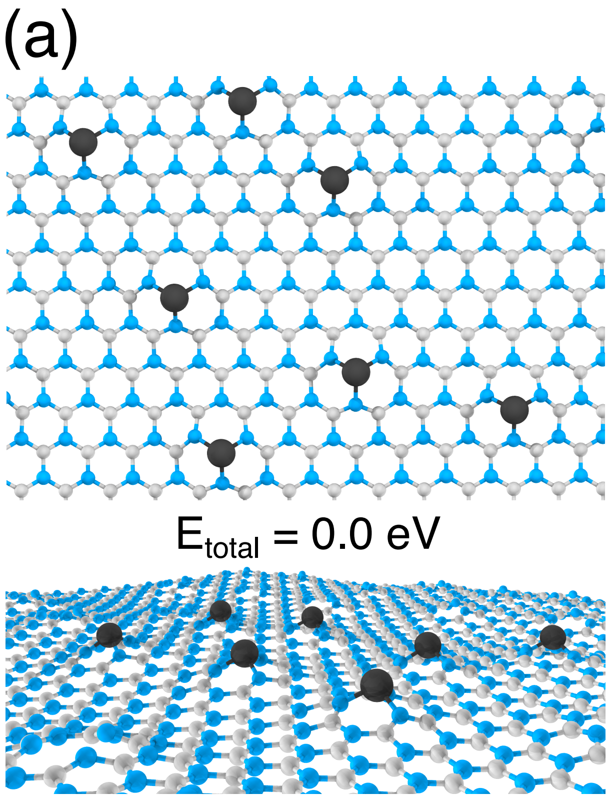

Figure S2: Relaxed structures of seven Pt atoms on defective *h*-BN (B-vacancies). The dispersed configuration is 19.70 eV lower in energy than the clustered form, making it more energetically favourable. Top-view (top panels) and side-view (bottom panels) images are shown.

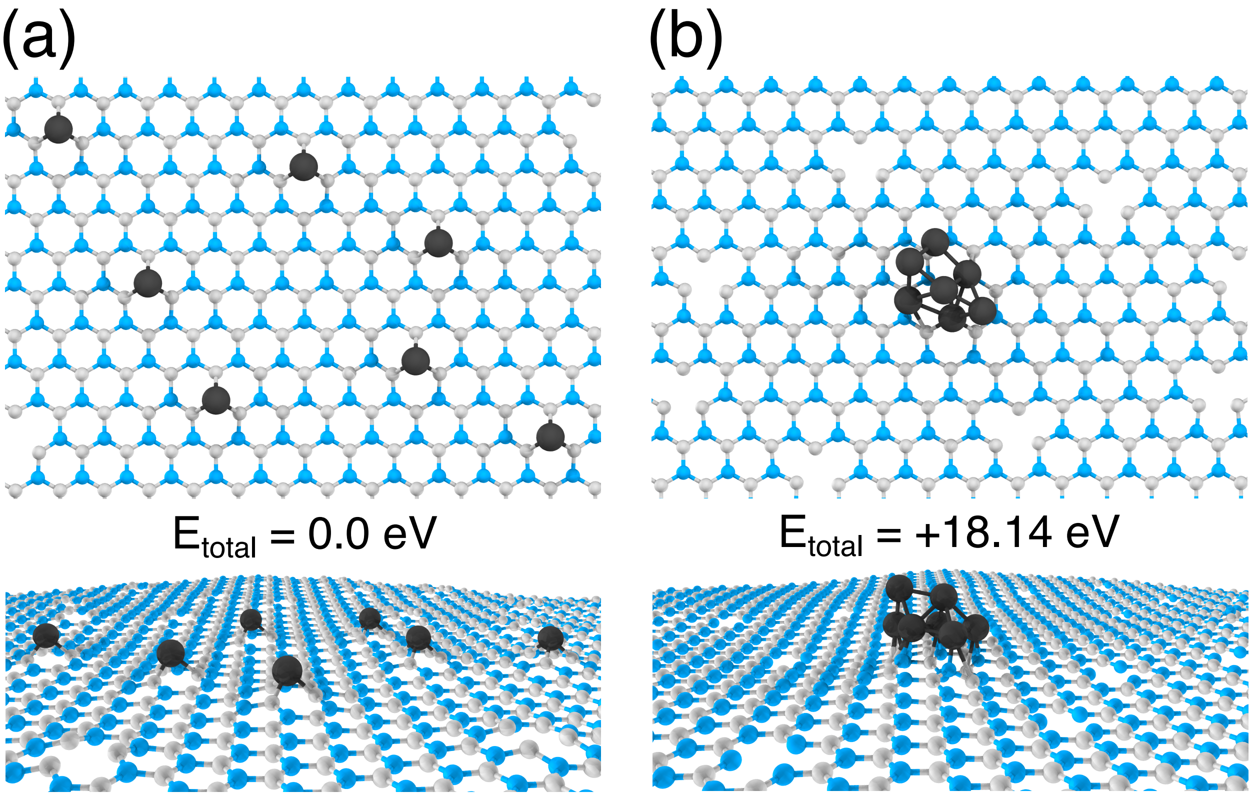

Figure S3: Relaxed structures of seven Pt atoms on defective *h*-BN (N-vacancies). The dispersed configuration is 18.14 eV lower in energy than the clustered form, making it more energetically favourable. Top-view (top panels) and side-view (bottom panels) images are shown.

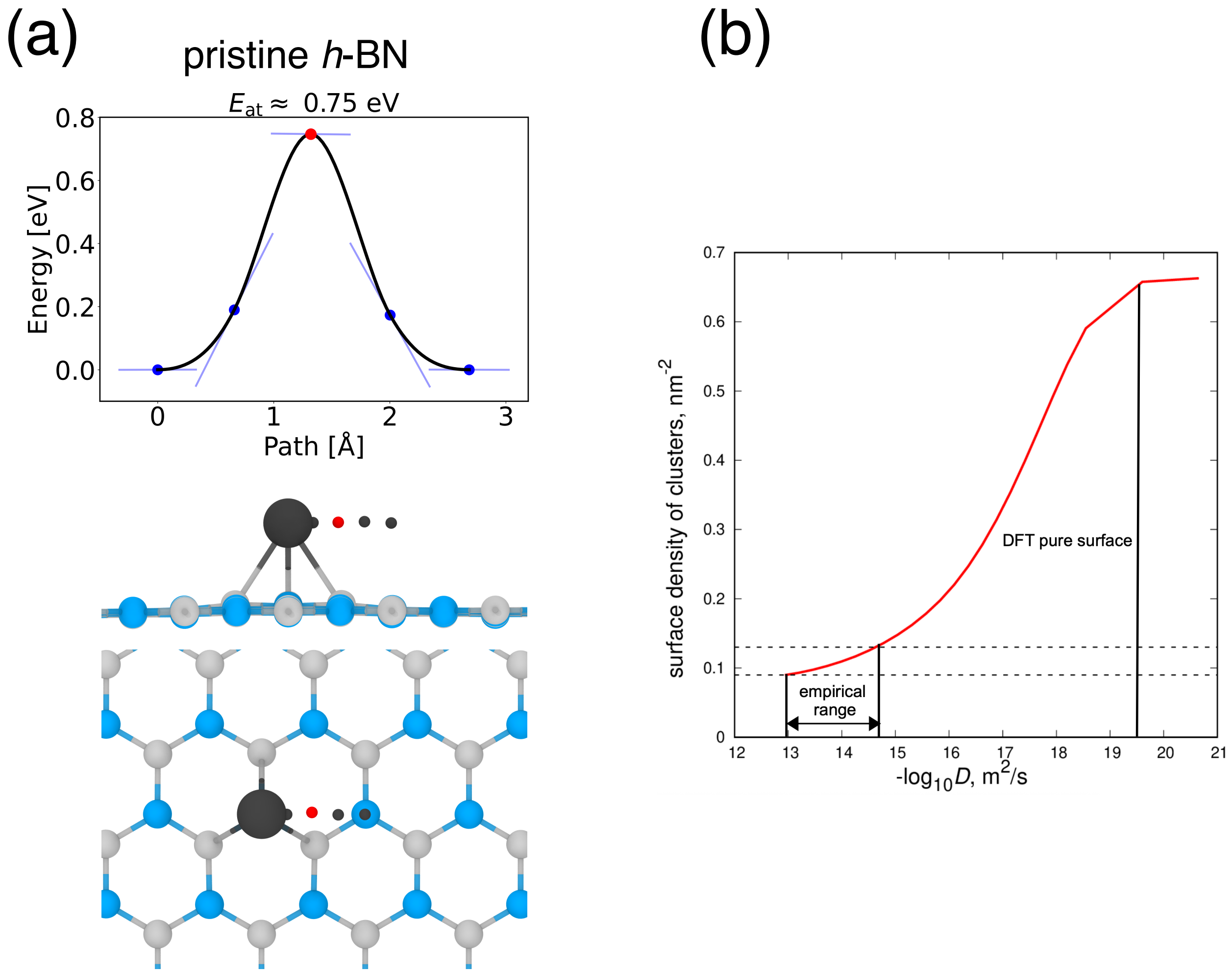

Figure S4: (a) DFT-based nudged elastic band calculation of the energy barrier for a Pt adatom on pristine *h*-BN. (b) red solid curve corresponds to the dependence of the surface density of Pt nanoclusters on the diffusion coefficient of Pt adatoms on *h*-BN, as predicted by the kinetic nucleation theory.[1] The parameters selected for the theoretical model were set up to match the experimental conditions, namely, the time of deposition, τ, was taken to be 1 second, temperature of 298K, the flow of Pt atoms $J = 1.8\,\mathrm{nm}^{-2}\cdot\mathrm{s}^{-1}$, and the initial concentration of point defects $N_d = 0.1\,\mathrm{nm}^{-2}$. Dashed horizontal lines indicate a range of the experimentally observed values of the surface density (0.11 ± 0.2) nm^{-2}, and solid vertical lines correspond to the experimental and DFT values of the diffusion coefficient.

S2: Volmer - Weber kinetic nucleation model in the presence of point defects

We denote the surface concentration of clusters containing i atoms formed in homogeneous nucleation as n_i, and clusters formed on the point defects are labelled as f_i. The time evolution of the nucleation process is described by the following set of differential equations:

$$\frac{dn_1}{dt} = J\left(1-\theta\right) - \alpha_1 J n_1 - 2k_1 n_1^2 - \sum_{i\geq 2} k_i n_1 n_i - \sum_{i\geq 0} k_i^d n_1 f_i, \tag{1}$$

$$\frac{dn_i}{dt} = k_{i-1} n_1 n_{i-1} - k_i n_1 n_i + \alpha_{i-1} J n_{i-1} - \alpha_i J n_i, \quad i \geq 2, \tag{2}$$

$$\frac{df_0}{dt} = -k_0^d n_1 f_0 - \alpha_0 J f_0, \tag{3}$$

$$\frac{df_i}{dt} = k_{i-1}^d n_1 f_{i-1} - k_i^d n_1 f_i + \alpha_{i-1} J f_{i-1} - \alpha_i J f_i, \quad i \geq 1, \tag{4}$$

where the fractional surface occupancy has the form

$$\theta = \sum_{i\geq 1} \alpha_i \left(n_i + f_i\right). \tag{5}$$

In these equations, n_1 corresponds to the surface concentration of mobile single metal atoms, f_0 is the concentration of point defects without attached metal atoms, and f_1 is the concentration of single metal atoms trapped by the point defects.

The first term in equation (1) corresponds to the flow of atoms to the surface followed by on-top attachment directly from the gas, formation of a dimer from two adatoms and, finally, the last two terms describe the lateral attachments of metal atoms to nanoclusters growing on a defect-free part of the surface and on point defects, respectively. Equations (1) - (4) are solved with the initial conditions $f_0\left(0\right) = N_d$, $n_i\left(0\right) = 0$ and $f_i\left(0\right) = 0$ for $i \geq 1$. If deposition occurs over time τ, then

the observed ratio of single atoms to nanoclusters can be estimated as

$$\mathrm{SA} : \mathrm{NC} = f_1 (\tau) \left\{ \sum_{i \geq 2} \left[n_i (\tau) + f_i (\tau) \right] \right\}^{-1} . \tag{6}$$

The kinetic parameters α_i, k_i, and k_i^d are time independent and can be defined in the way described in ref.[1] and the main text of the paper.

References

(1) Popov, I.; Ghaderzadeh, S.; Kohlrausch, E. C.; Norman, L. T.; Slater, T. J. A.; Aliev, G. N.; Alhabeadi, H.; Kaplan, A.; Theis, W.; Khlobystov, A. N.; Fernandes, J. A.; Besley, E. Chemical Kinetics of Metal Single Atom and Nanocluster Formation on Surfaces: an Example of Pt on Hexagonal Boron Nitride. *Nano Lett.* **2023**, *23*, 8006–8012.
